# Supplementary material for: Amine‐to‐Azide Conversion on Native RNA via Metal‐Free Diazotransfer Opens New Avenues for RNA Manipulations
Source: Angew Chem Weinheim Bergstr Ger. 2021 Feb 18;133(13):7046–50. doi: 10.1002/ange.202015034 (PMC10947191; doi:10.1002/ange.202015034)
Supplement: Supplementary file 1 — Supplementary [file ANGE-133-7046-s001.pdf]

Angewandte Chemie
Eine Zeitschrift der Gesellschaft Deutscher Chemiker

Supporting Information

Amine-to-Azide Conversion on Native RNA via Metal-Free Diazotransfer Opens New Avenues for RNA Manipulations

*Olga A. Krasheninina, Julia Thaler, Matthias D. Erlacher, and Ronald Micura**

ange_202015034_sm_miscellaneous_information.pdf

Supporting information

Contents

Methods S2

1. RNA solid-phase synthesis, deprotection, and purification S2
2. RNA mass spectrometry S3
3. Amine-to-azido conversion on RNA through diazotransfer reaction S3
   3.1. General S3
   3.2. Preparation of fluorosulfuryl azide (FSO_2N_3) S4
   3.3. Diazotransfer reaction on RNA using FSO_2N_3 S4
4. Synthesis of *N*-formyl-*L*-methionine 2-(diphenylphosphino)phenyl ester S5
5. Labeling of RNAs with biotin-PEG4-alkyne by copper(I)-catalyzed cycloaddition S8
6. RNA-peptide conjugates by traceless Staudinger ligation S8
7. Pull-down of tRNAs containing nucleotide modifications with primary amino groups S9
8. Northern blotting assay for tRNA identification S9

Supporting Figures S11

Supporting Figure S1 S11
Supporting Figure S2 S12
Supporting Figure S3 S13
Supporting Figure S4 S14
Supporting Figure S5 S15
Supporting Figure S6 S16
Supporting Figure S7 S17
Supporting Figure S8 S18

Supporting Tables S19

Supporting Table S1 S19
Supporting Table S2 S20

References S21

Methods

1. RNA solid-phase synthesis, deprotection, and purification

All RNAs were assembled on an ABI392 synthesizer at 1 µmol scale using 2'-*O*-TOM or 2'-*t*BDMS nucleoside phosphoramidites (ChemGenes), polystyrene supports (GE Healthcare, Custom Primer Support™, 80 µmol g^{-1}; PS 200 and Primer Support™ 5G, 300 µmol g^{-1}) and CPG supports (ChemGenes, CPG 1000 Å, 30-40 µmol g^{-1}). The 3'-amino-3'-deoxyadenosine-functionalized supports (rA3'-NH-Phe, rA3'-NH-Ile, rA3'-NH-Val and rA3'-NH-Gly) for the automated solid-phase synthesis of RNA-amino acid conjugates were prepared according to [1].

Standard RNA synthesis cycle: 1) detritylation with dichloroacetic acid/1,2-dichloroethane (4/96) (120 s); 2) coupling with phosphoramidites in acetonitrile (0.1 M) and benzylthiotetrazole in acetonitrile (0.3 M) (180 s); 3) capping with Cap A: phenoxyacetic anhydride (0.2 M) in dry THF and Cap B: *N*-methyl imidazole and *sym*-collidine (0.2 M each) in dry THF (2 x 15 s, Cap A/Cap B, 1:1); 4) oxidation with iodine (20 mM) in tetrahydrofuran (THF)/pyridine/H_2O (40/1/9) (60 s).

The modified nucleoside building block of 5'-dimethoxytrityl-5-(trifluoroacetylamino)methyl-uridine-2'-*O*-triisopropylsilyloxymethyl-3'-[(2-cyanoethyl)-(*N*,*N*-diisopropyl)]-phosphoramidite was incorporated using modified synthesis cycles with longer coupling time (up to 6 min).

To remove cyanoethyl groups and the *N*-Fmoc group of the assembled aminoacyl-3'-*NH*-RNAs, the support (~25 mg) was kept in the synthesis cartridge and rinsed with 20 mL of 20% piperidine in acetonitrile followed by 20 mL of acetonitrile. Then, for base deprotection and cleavage from the support, the beads were transferred into an Eppendorf tube and equal volumes of CH_3NH_2 in ethanol (8 M, 650 µL) and CH_3NH_2 in H_2O (40%, 650 µL) were added. The mixture was kept at room temperature for 8 hours. After that, the supernatant was filtered and evaporated to dryness in a SpeedVac concentrator.

For base deprotection and cleavage from the support of 5-aminomethyl uridine containing RNAs, the support-bound protected RNAs were treated with a mixture of 40% aqueous methylamine and 30% aqueous ammonia (600 µL, 1:1 v/v) in a screw-cap vial at 65 °C for 15 minutes. Then, the supernatant was filtered and reaction mixture were evaporated to dryness in the SpeedVac.

The 2'-*O*-silyl (TOM or *t*BDMS) protecting groups were removed by incubation of oligoribonucleotides in a mixture of *N*-methyl-2-pyrrolidone (100 µL) and 1 M TBAF trihydrate in THF (1000 µL) at 37 °C for 16 h. The reaction was quenched by addition of 1 M triethylammonium acetate, pH 7.4 (1000 µL) and then concentrated to approximately 0.5 to 1.0 mL. The resulting mixture was desalted on a C18 Sep-Pak® cartridge according to a protocol recommended by the manufacturer (Waters Corporation). The crude oligonucleotide products were evaporated and stored at -20 °C.

Quality assessment of all the RNAs was performed using anion-exchange HPLC on a Dionex DNAPac PA-100 column (4 x 250 mm); conditions: flow rate 1 mL min^{-1}; eluent A: 25 mM Tris-HCl, pH 8.0, 6 M urea, eluent B: 500 mM $NaClO_4$, 25 mM Tris-HCl, pH 8.0, 6 M urea; gradient: 0-60% B in A, in 45 minutes; 60 °C, UV detection at 260 nm.

The desired oligoribonucleotides were purified and isolated by semi-preparative anion-exchange HPLC on a Dionex DNAPac PA-100 column (9 x 250 mm). Conditions: flow rate 2 mL min^{-1}; for eluents and

UV detection see above; the gradient was optimized according to the length of the RNA and typically Δ15% B in 30 min.

The fractions corresponding to a product were desalted using the C18 Sep-Pak® cartridges (Waters Corporation). The quality of the product was analyzed by anion-exchange HPLC and reversed-phase LC-ESI MS. Sequences and mass spectrometric data for all the obtained RNAs are shown in Supporting Table S1.

2. RNA mass spectrometry

All experiments were performed on a Finnigan LCQ Advantage MAX ion trap instrumentation connected to a Thermo Fisher Ultimate 3000 HPLC system. RNAs were analyzed in the negative-ion mode with a potential of −4 kV applied to the spray needle. LC: Sample (200 pmol RNA dissolved in 30 µL of 20 mM ethylenediamine tetraacetic acid (EDTA) solution; average injection volume: 30 µL); column (Waters XTerraMS, C18, 2.5 µm; 1.0 × 50 mm) at 21°C; flow rate: 0.1 mL/min; eluant A: 8.6 mM triethylamine (TEA), 100 mM 1,1,1,3,3,3-hexafluoroisopropanol in H_2O (pH 8.0); eluant B: methanol; gradient: 0–100% B in A within 30 min; UV detection at 254 nm.

3. Amine-to-azido conversion on RNA through diazotransfer reaction

3.1. General

Materials

Reagents were purchased in the highest available quality from commercial suppliers (Merck / Sigma-Aldrich, ChemGenes) and used without further purification. All reactions were carried out under argon atmosphere, unless otherwise noted. Analytical thin-layer chromatography (TLC) was performed on Macherey-Nagel Polygram® SIL G/UV254 plates. Silica gel 60 (0.04 – 0.06 mm) for column chromatography was purchased from Macherey-Nagel. 1-(Fluorosulfuryl)-2,3-dimethyl-1*H*-imidazol-3-ium trifluoromethanesulfonate was purchased from Apollo Scientific Ltd.

NMR measurements of compounds

^{1}H, ^{13}C, and ^{31}P spectra were recorded on a Bruker UltrashieldTM 400 Plus spectrometer. Chemical shifts (δ) are reported relative to tetramethylsilane (TMS), referenced to the residual solvent signal (DMSO-d6: 2.50 ppm for ^{1}H and 39.52 ppm for ^{13}C spectra; $CDCl_3$: 7.26 ppm for 1H and 77.16 ppm for ^{13}C spectra). The following abbreviations were used to denote multiplicities: s = singlet, d = doublet, t = triplet, q = quadruplet, m = multiplet, b = broad. Signal assignments are based on ^{1}H-^{1}H-COSY, ^{1}H-^{13}C-HSQC, ^{1}H-^{13}C-HMBC experiments.

High-resolution mass spectrometry of compounds

High resolution mass spectra were recorded in positive ion mode on a Thermo Scientific Q Exactive Orbitrap, ionized via electrospray at 3.7 kV spray voltage.

3.2. Preparation of fluorosulfuryl azide (FSO_2N_3)

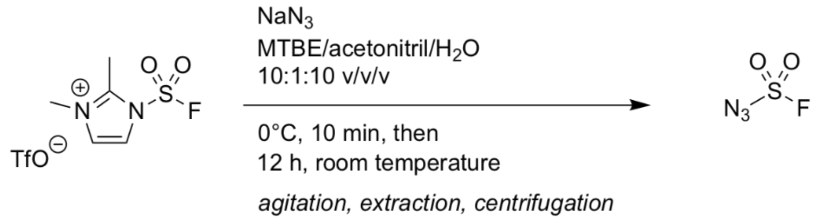

The diazotizing reagent, fluorosulfuryl azide (FSO_2N_3), was essentially prepared according to the procedure described in [2] and optimized as described in the following. To a stirred biphasic system of aqueous NaN_3 solution (97.5 mg, 1.50 mmol, 3 ml) and MTBE (3 mL) in a loosely sealed plastic bottle, cooled in an ice-water bath, was rapidly added a solution of 1-(fluorosulfuryl)-2,3-dimethyl-1*H*-imidazol-3-ium trifluoromethanesulfonate (0.5 g, 1.52 mmol; CAS 2179072-33-2)) in acetonitrile (0.25 mL). The reaction was stirred vigorously for 10 min at 0 °C and then rested at room temperature for 30 min. The organic phase containing FSO_2N_3 was separated from the aqueous phase, and it was kept in a loosely sealed plastic bottle at room temperature. After the 12-hour resting period, the organic phase was transferred to Eppendorf ultracentrifuge 2 ml tubes and centrifuged at 13,400 rpm for 15 min, to facilitate separation of the phases. The colorless solution of FSO_2N_3 in MTBE was separated from the pink residual aqueous phase and was used for the diazotransfer reaction directly without further purifications. The concentration and yield of the FSO_2N_3 solution were measured by ^{19}F NMR as described in [2], giving the values of 450 to 480 mM and ~90%, respectively.

3.3. Diazotransfer reaction on RNA using FSO_2N_3

For the diazotransfer reaction, an equivalent of RNA containing a primary amino group (final concentration in a range of 20-50 µM) was dissolved in 0.1 M $NaHCO_3$, pH 8.3 (100 µL) and DMF (20 µL), after that two equivalents of FSO_2N_3 in MTBE (90 to 210 µL) were added, and the mixture was thoroughly mixed (1.400 rpm) for 20 min at room temperature. Then, to facilitate separation of the phases, the reaction mixture was centrifuged at 13,400 rpm for 10 min. The colorless organic phase was removed from residual aqueous phase containing the RNA products. The azide-modified oligoribonucleotides were precipitated with 2% $NaClO_4$ in acetone (1000 µL), the resulting pellet was washed with acetone (600 µL) and air-dried. According to their AE-HPLC profiles, the conversion for all the reactions was >90% (see Supporting Table 1, Figure 2 and Supporting Figure 3).

4. Synthesis of *N*-formyl-*L*-methionine 2-(diphenylphosphino)phenyl ester (*PPh₃OfMet*)

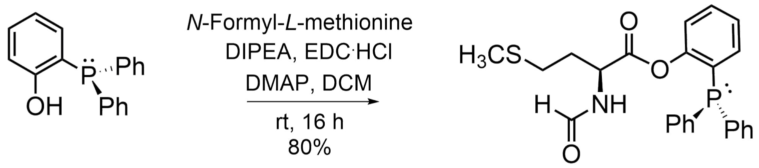

$C_{18}H_{16}PO$ [278.29]

PPh₃OfMet $C_{24}H_{24}PNSO_3$ [437.49]

To a solution of 2-(diphenylphosphino)phenol (417 mg, 1.5 mmol) and *N*-formyl-*L*-methionine (300 mg, 1.7 mmol) in CH_2Cl_2 (4 mL) were added *N*-(3-dimethylaminopropyl)-*N'*-ethylcarbodiimide (263.5 mg, 1.7 mmol), *N,N*-diisopropylethylamine (400 μL), and *N,N*-dimethyl-4-aminopyridine (~5 mg, catalytic amount) at room temperature. After being stirred at room temperature for 16 h, the reaction mixture was directly purified by column chromatography on silica gel (CH_2Cl_2/EtOAc, 10:1 to 1:1) to give phosphinophenol ester (526 mg, 1.2 mmol, 80%): white foam; TLC (CH_2Cl_2/EtOAc, 10:1, v/v): R_f 0.2. ^1H NMR (400 MHz, DMSO-*d6*, δ, ppm): 1.73-1.87 (m, 1H, -S-H₂C-***H***HC-), 1.96 (s, 3H, ***H₃***C-S-), 1.89-2.01 (m, 1H, -S-H₂C-H***H***C-), 2.38-2.48 (m, 2H, H₃C-S-***H₂***C-), 4.50-4.59 (m, 1H, -***H***C*-), 6.73-6.82 (m, 1H, Ar), 7.14-7.30 (m, 6H, Ar), 7.37-7.53 (m, 7H, Ar), 8.17 (s, 1H, -***H***C=O), 8.70 (d, *J* = 7.53, 1H, -***H***N-). ^{13}C NMR (100.62 MHz, DMSO-*d6*, δ, ppm): 14.85, 29.80, 30.25, 50.15, 123.02, 127.13, 129.31, 129.38, 130.02, 131.09, 131.75, 133.71, 133.79, 133.84, 133.91, 133.99, 135.42, 135.72, 153.02, 153.21, 161.83, 170.19. ^{31}P NMR (161.97 MHz, DMSO-*d6*, δ, ppm): -18.42, -19.03 (diastereomers). HR-ESI-MS (m/z, $C_{24}PNSO_3H_{24}$): $[M+H]^+$ calcd.: 438.50, found: 438.13; $[M+Na]^+$ calcd.: 460.48, found: 460.11.

^{1}H NMR (400 MHz, DMSO-*d6*) of ***PPh₃OfMet***:

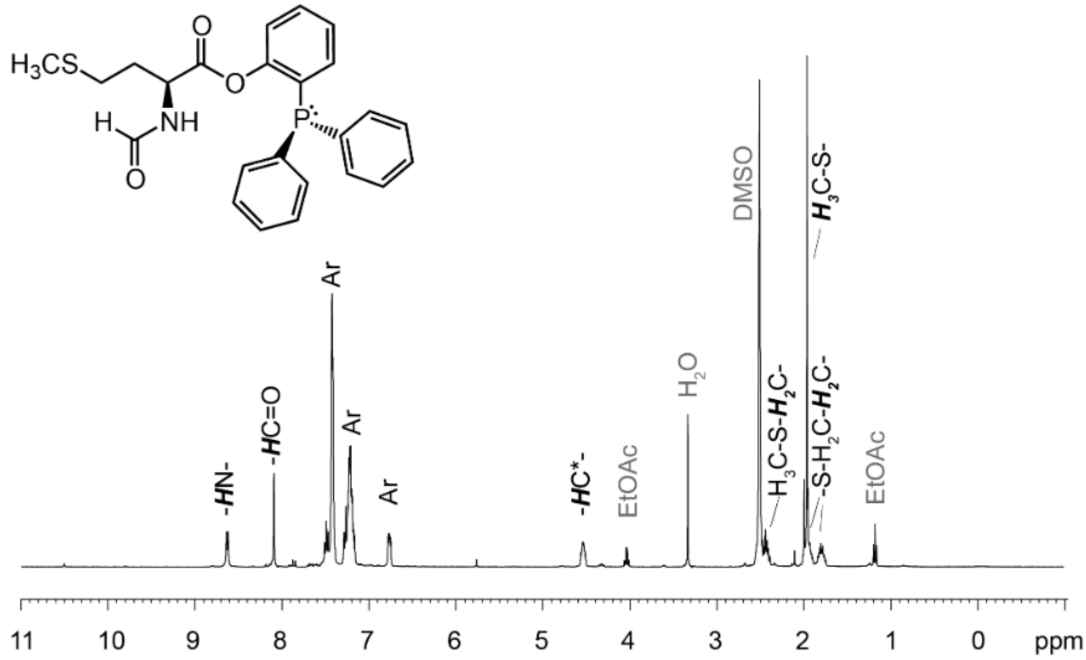

^{13}C NMR (100 MHz, DMSO-*d6*) of ***PPh₃OfMet***:

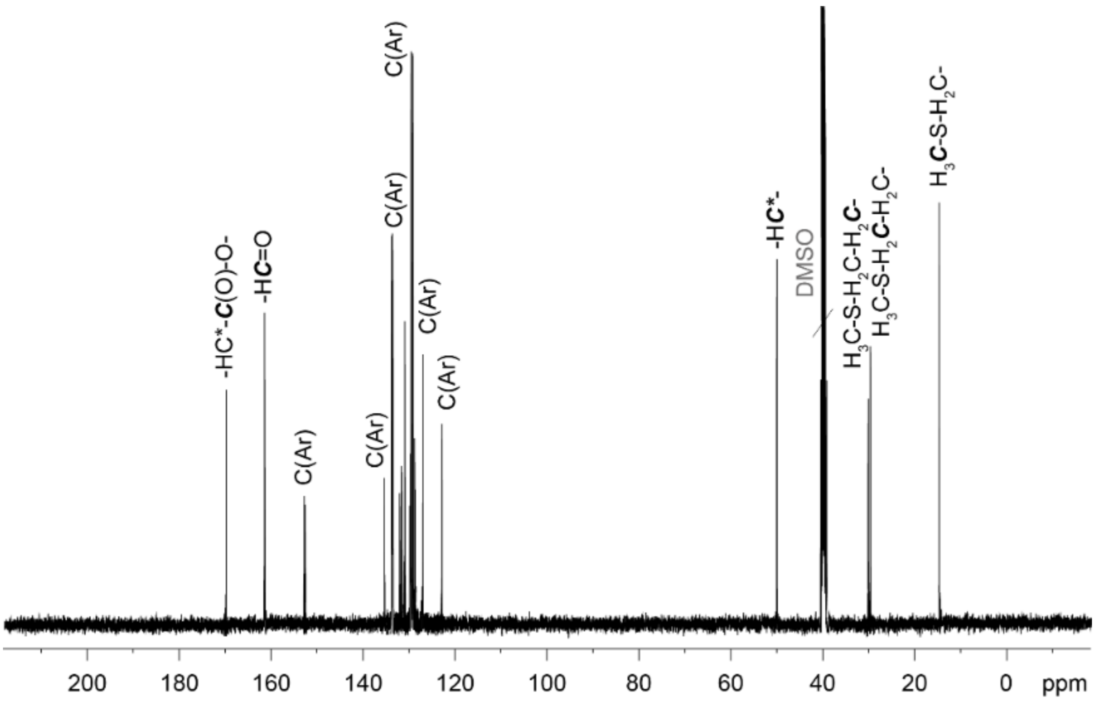

^{31}P NMR (162 MHz, DMSO-*d6*) of ***PPh$_3$OfMet***:

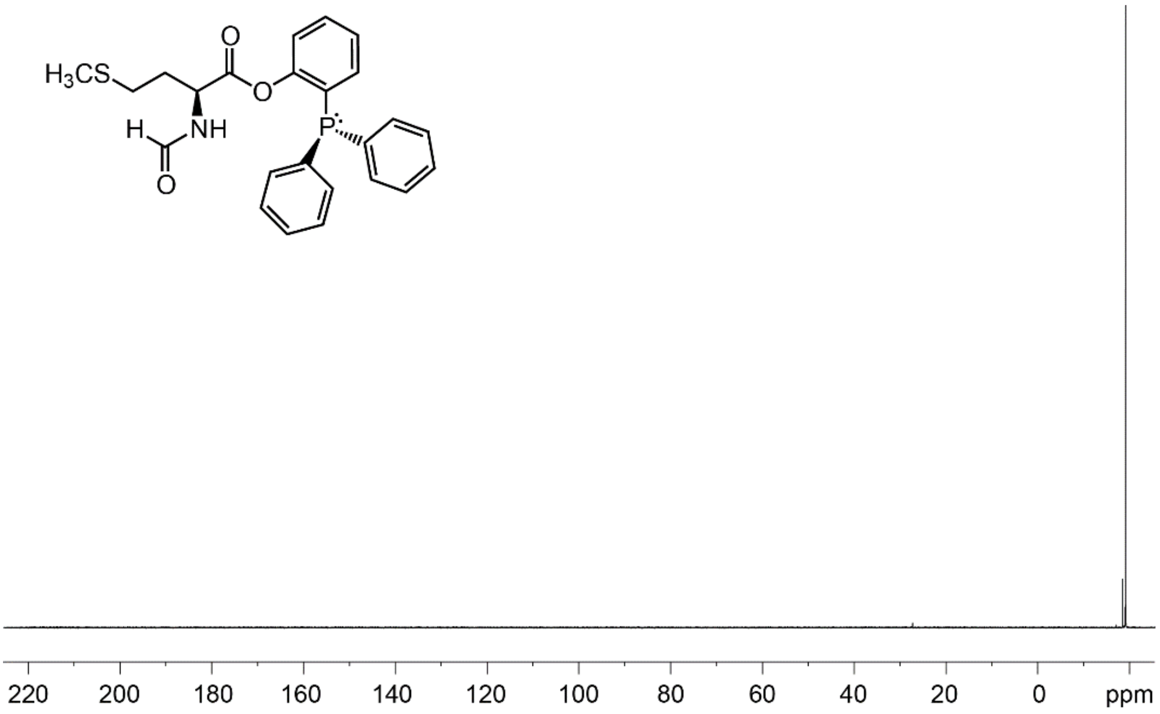

HR-MS of ***PPh$_3$OfMet***:

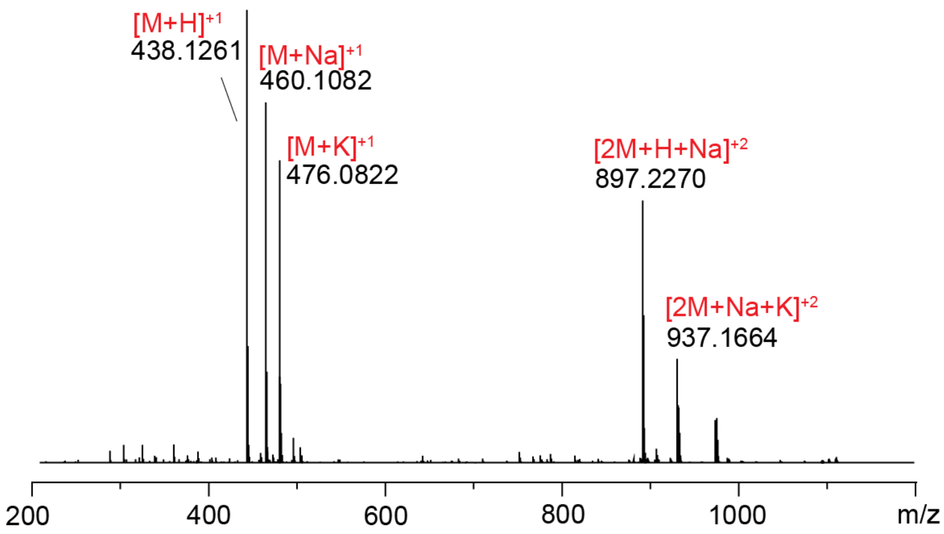

5. Labeling of RNAs with biotin-PEG4-alkyne by copper(I)-catalyzed cycloaddition (CuAAC)

For the CuAAC, the copper(I)-catalyst was generated *in situ* from a complex of $CuSO_4$ and water-soluble tris(3-hydroxypropyltriazolylmethyl)amine (THPTA ligand) [3] using freshly prepared solution of sodium ascorbate as a reducing agent. In brief, a 70 mM solution of THPTA ligand (50 µL) was mixed with a 0.1 M solution of $CuSO_4 \cdot 5H_2O$ (5 µL), purged with a slow flow of argon for 1 min, and mixed with sodium ascorbate (50 µL; 1 M aqueous solution). This solution was added to the degassed mixture of annealed azido modified oligoribonucleotide (2-6 nmol in 20 µL of deionized H_2O) and biotin-PEG4-alkyne (0.4 mg, ~1 µmol) in DMF (20 µL). The reaction mixture was degassed again and kept at room temperature for 1 h. After that, the oligoribonucleotides were precipitated with 2% $NaClO_4$ in acetone (1000 µL), the resulting pellet was washed with acetone (600 µL) and air-dried. The oligoribonucleotide material was dissolved in water (400 µL), and Vivaspin 500 Centrifugal Concentrators (Sartorius) were used to remove reagents from oligonucleotide material. According to the AE-HPLC profiles, a conversion for all of the reactions was in a range of 78-98 % (see Figure 3A and Supporting Figure 4, Supporting Table 1).

6. RNA-peptide conjugates by traceless Staudinger ligation

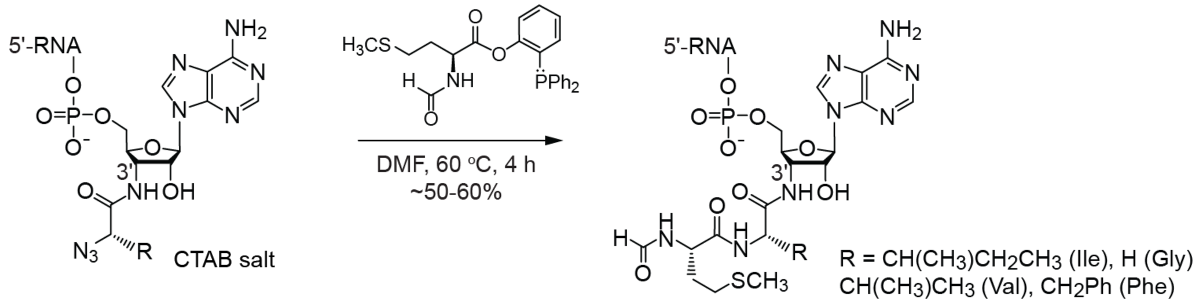

For the traceless Staudinger ligation, azido-modified oligonucleotide was converted to a form soluble in organic solvents. For this purpose, an oligoribonucleotide (2-6 nmol in 50-100 µL of deionized H_2O) was precipitated by 8% cetyltrimethylammonium bromide (CTAB), with a few portions of 2 µL each. After addition of each CTAB portion, the mixture was vortexed and centrifuged (2 min, 13400 rpm). The precipitation by CTAB was continued until the solution ceased to cloud with the addition of CTAB reagent. Then, the remaining water was removed, and the pellet was dissolved in DMF (100 µL) at 60 °C. The ***PPh₃OfMet*** ester (4.4 mg, 10 µmol) was added to the resulting solution of the cetyltrimethylammonium salt of the azido modified oligoribonucleotide in DMF. The reaction mixture was stirred for 4 h at 60 °C. After that, the oligoribonucleotide was precipitated with 2% $NaClO_4$ in acetone (1000 µL), the resulting pellet was washed with acetone (600 µL) and air-dried. According to the AE-HPLC profiles, a conversion for the reaction varied from ~60-86 % depending on the RNA conjugate (see Figure 3B and Supporting Figure 4, Supporting Table 1).

7. Pull-down of *E. coli* tRNAs containing nucleotide modifications with primary amino groups

Labeling. Total tRNA from *E. coli* (~1 mg, Sigma-Aldrich) was dissolved in deionized H_2O (88 µL) and DMF (20 µL), incubated at 80 °C for 3 min, and then immediately chilled on ice. A solution of 1 M $NaHCO_3$ (12 µL, to final concentration of 0.1 M, pH 8.3) was added to the mixture. Thereafter, FSO_2N_3 in MTBE (200 µL) was added, and the mixture was thoroughly mixed (1.400 rpm) for 20 min at room temperature. The reaction mixture was centrifuged at 13,400 rpm for 10 min. The colorless organic phase was removed and the tRNA pool was precipitated from the residual aqueous phase by adding 3 M sodium acetate (10 µL, pH 5.2) and ice-cold absolute ethanol (600 µL), the resulting pellet was washed with ice cold 70 % ethanol (400 µL) and air-dried. The following labeling of the tRNA pool with desthiobiotin-PEG4-alkyne (Jena Bioscience) was performed according to the procedure described above in Section 5. After that, the Vivaspin 500 Centrifugal Concentrators were used to remove reagents from the tRNA material.

For analysis of the complex reaction mixture after the diazotransfer and CuAAC reactions, the *E.coli* tRNAs were directly labeled with commercial alkyne-modified 5-carboxytetramethylrhodamine dye (F545) and subjected to denaturing polyacrylamide gel electrophoresis (in 10 % PAAG (19:1), 8 M urea). A fluorescent band of the F545-labeled tRNAs was observed with the expected electrophoretic mobility similar to the unmodified tRNA reference, consistent with successful labeling of the azido-modified tRNAs by the CuAAC reaction without RNA degradation (Supporting Figure S7).

Immobilization. The Streptavidin Magnetic Particles (magnetic beads, Sigma-Aldrich) were thoroughly mixed and a 200 µL aliquot of the beads was taken for capturing of the biotinylated tRNA species. The supernatant was removed and the beads were washed twice with a solution of 100 mM NaOH and 50 mM NaCl (300 µL), then washed once with a solution of 100 mM NaCl (300 µL), followed by washing twice with a binding buffer consisting of 10 mM Tris-HCl, pH 7.5, 1 mM EDTA, 2 M NaCl (200 µL). After that, the binding buffer (200 µL) was added and the beads were resuspended. The labeled tRNA pool was added to the suspension of the beads, the mixture was gently mixed by pipetting, and incubated at 25 °C for 30 min. The supernatant was removed and the beads were washed twice with 200 µL of 20 mM Tris-HCl (pH 7.5).

Non-denaturing elution. A solution of biotin (Sigma-Aldrich) (~5 mg/mL) was added to the tRNA modified beads. The suspension was mixed by pipetting and incubated at room temperature for 30 min with gentle shaking to prevent sedimentation of the beads. Next, the beads were centrifuged at 2000×g for 30 s, and the supernatant was collected. The elution was repeated three more times. After that, the Vivaspin 500 Centrifugal Concentrators were used to remove reagents from the collected supernatant containing tRNA material. The tRNA pool was precipitated from the residual aqueous phase by adding a solution of 3 M sodium acetate (10 µL, pH 5.2) and ice cold absolute ethanol (600 µL), the resulting pellet was washed with ice cold 70% ethanol (400 µL) and air-dried.

For schematic representation of the tRNA enrichment procedure and the cloverleaf structures of all the *E. coli* tRNAs containing acp^3U47 see Figure 4 and Supporting Figure S6, respectively.

8. Northern blotting assay to identify tRNA targets having primary amino groups

The tRNA pool obtained after the tRNA enrichment procedure (~20 µg) was heated at 80 °C in a mixture containing 8 M urea, 0.05% xylene cyanol FF and 0.05% bromophenol blue for 2 min, then cooled to room temperature, and loaded on a denaturing 10% polyacrylamide gel (19:1, 8 M urea; ~2 µg/pocket).

The transcribed $tRNA^{Lys}$ was used in the experiment as an RNA ladder. Following standard capillary transfer procedures, tRNA pool was transferred in 0.5x TBE buffer (0.05 M Tris base, 0.05 H_3BO_3, and 1 mM EDTA, pH 8.3) to a positively charged nylon membrane (Hybond®-N+ hybridization membrane). Subsequently, tRNAs were immobilized on the membrane by irradiation in the BioDocAnalyze (Biometra) for 2 min. After 60 min of prehybridization in a hybridization buffer (1 M sodium phosphate buffer pH 6.2, 7 % SDS), a 5′-^{32}P-labeled probe (prepared with [gamma -^{32}P]ATP and T4 polynucleotide kinase according to standard procedures) complementary to a variable region of the respective tRNAs from *E. coli* (20 pmol, see sequences in Supporting Table 2) was added and the blot was incubated overnight at 42 °C. Next, the blot was washed in a washing buffer I (0.1 % SDS, 0.6 M NaCl, 0.06 M trisodium citrate, pH 7.0) for 3 min at 25 °C, then in a washing buffer II (0.1 % SDS, 0.03 M NaCl, 3 mM trisodium citrate, pH 7.0) for 2 min and rinsed in water. The membrane was air-dried and placed in an X-ray cassette, where it was exposed for 24 h. Visualization of bands was performed using Typhoon FLA 9500 phosphorimager (GE Healthcare).

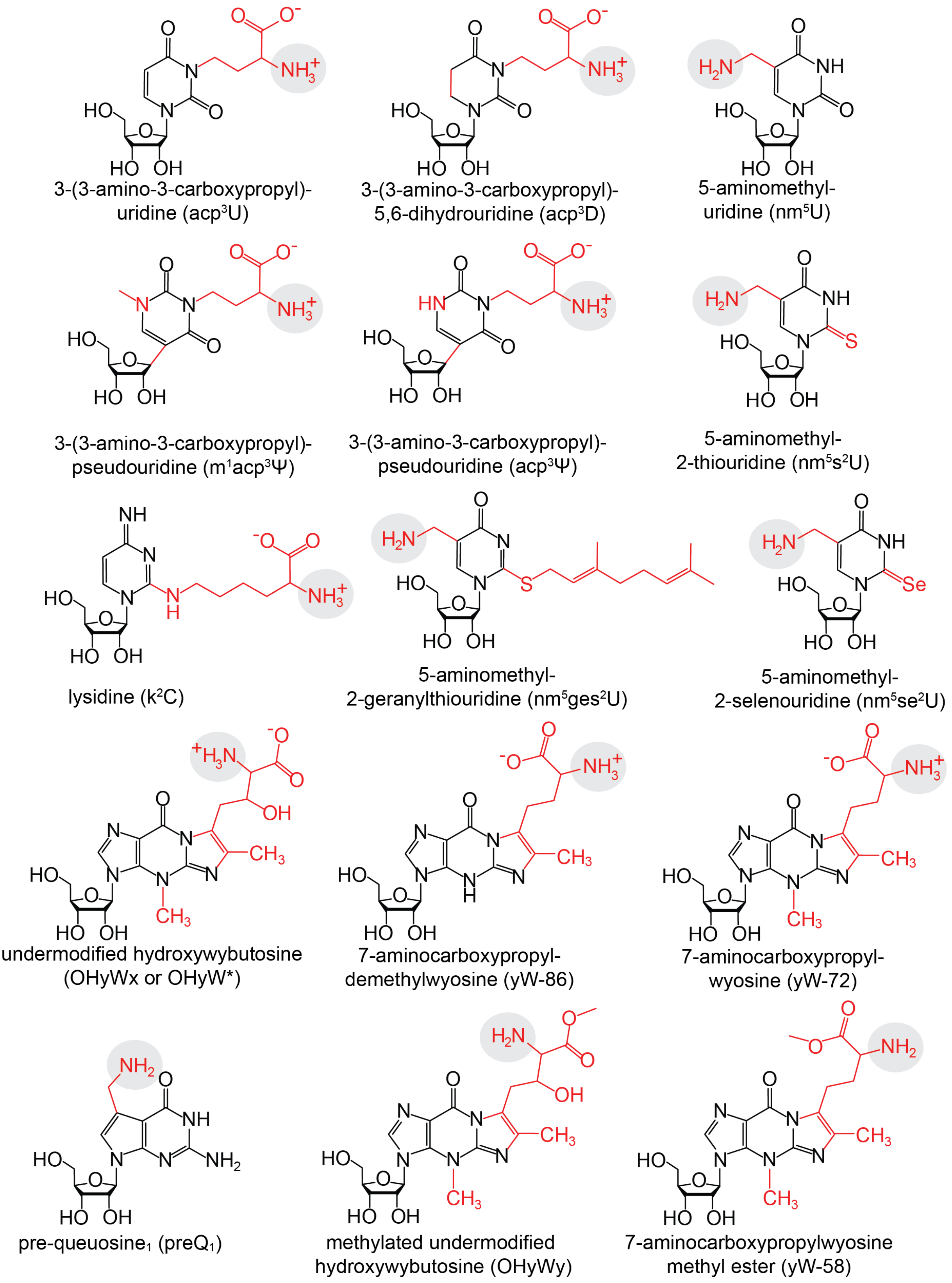

Supporting Figure S1. Diversity of known natural RNA modifications having a primary aliphatic amino group and found in rRNAs and tRNAs [4].

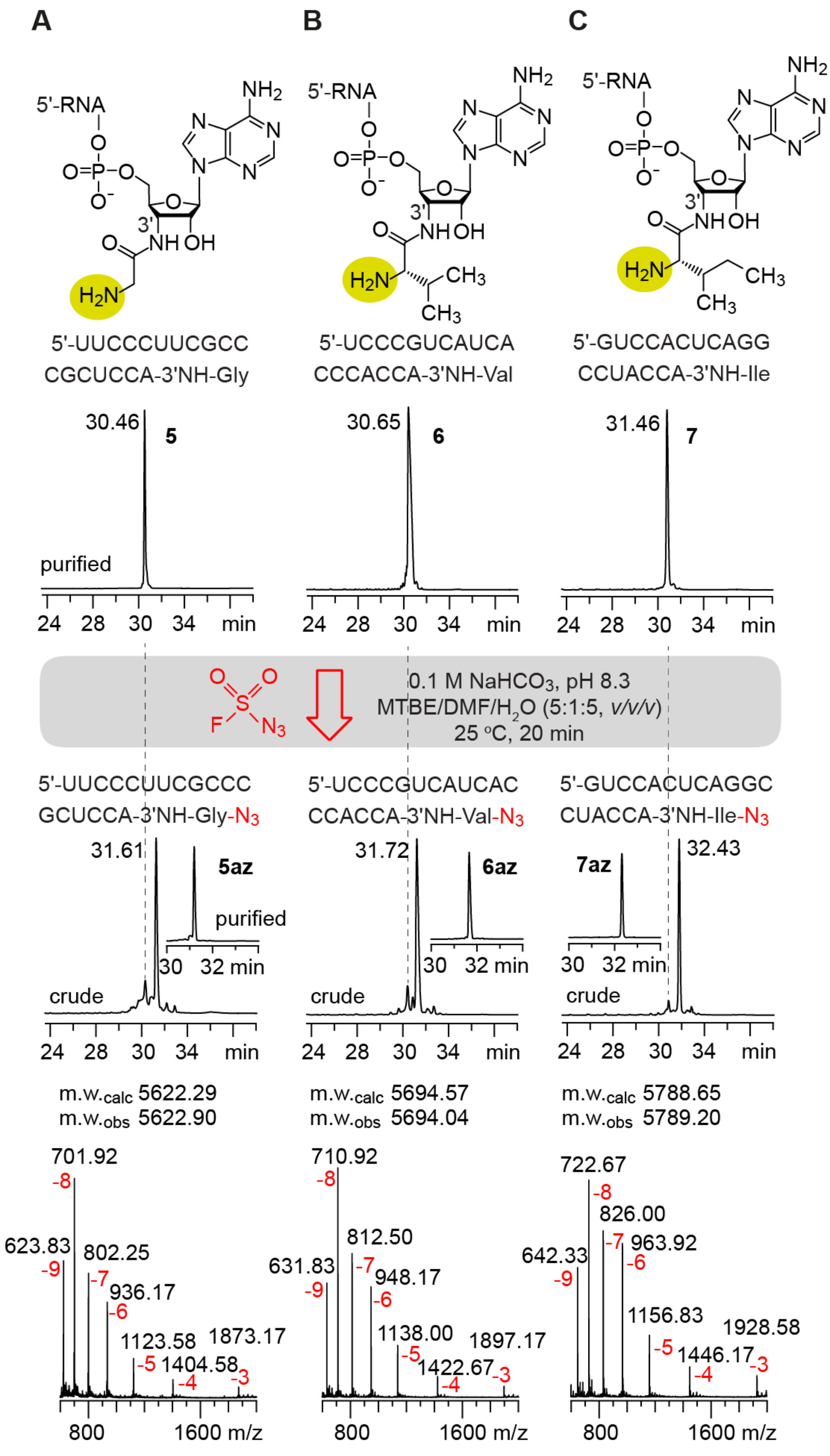

Supporting Figure S2. Diazotransfer reaction between aminoacyl-3'-*NH*-RNAs and FSO_2N_3. Anion-exchange HPLC analysis of the reaction mixtures and mass spectrometric characterization of the corresponding azidoacyl-3'-*NH*-RNA products. (**A**) 18 nt glycyl-tRNA^{Gly} terminal fragment, **5** and **5az**; (**B**) 18 nt L-valyl-tRNA^{Val} terminal fragment, **6** and **6az**; (**C**) 18 nt L-isoleucyl-tRNA terminal fragment, **7** and **7az**. For HPLC and MS conditions see Methods.

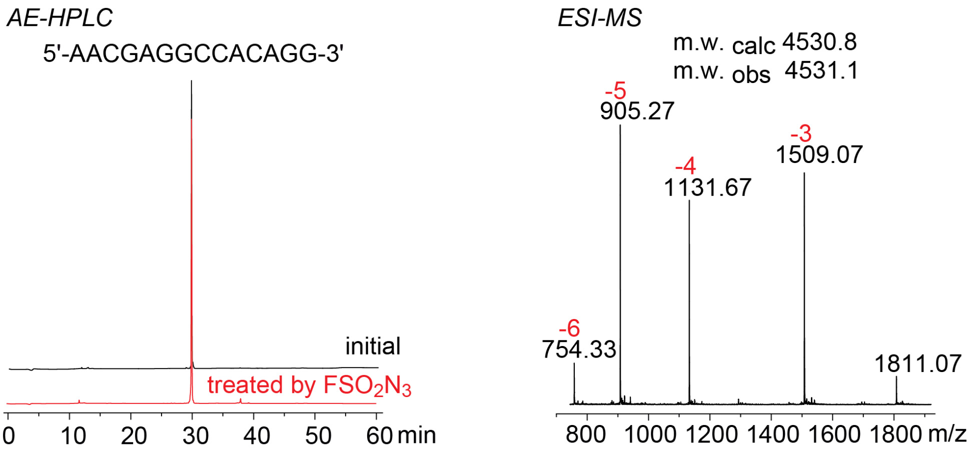

Supporting Figure S3. Anion-exchange HPLC and mass spectrometric analysis of a reference RNA (5'-AACGAGGCCACAGG-3') having no aliphatic primary amino groups after being exposed to the optimized diazotransfer reaction conditions. The molecular weight is calculated for the intact reference RNA. The RNA is stable under these conditions and no reaction occurs at the nucleobase amino groups.

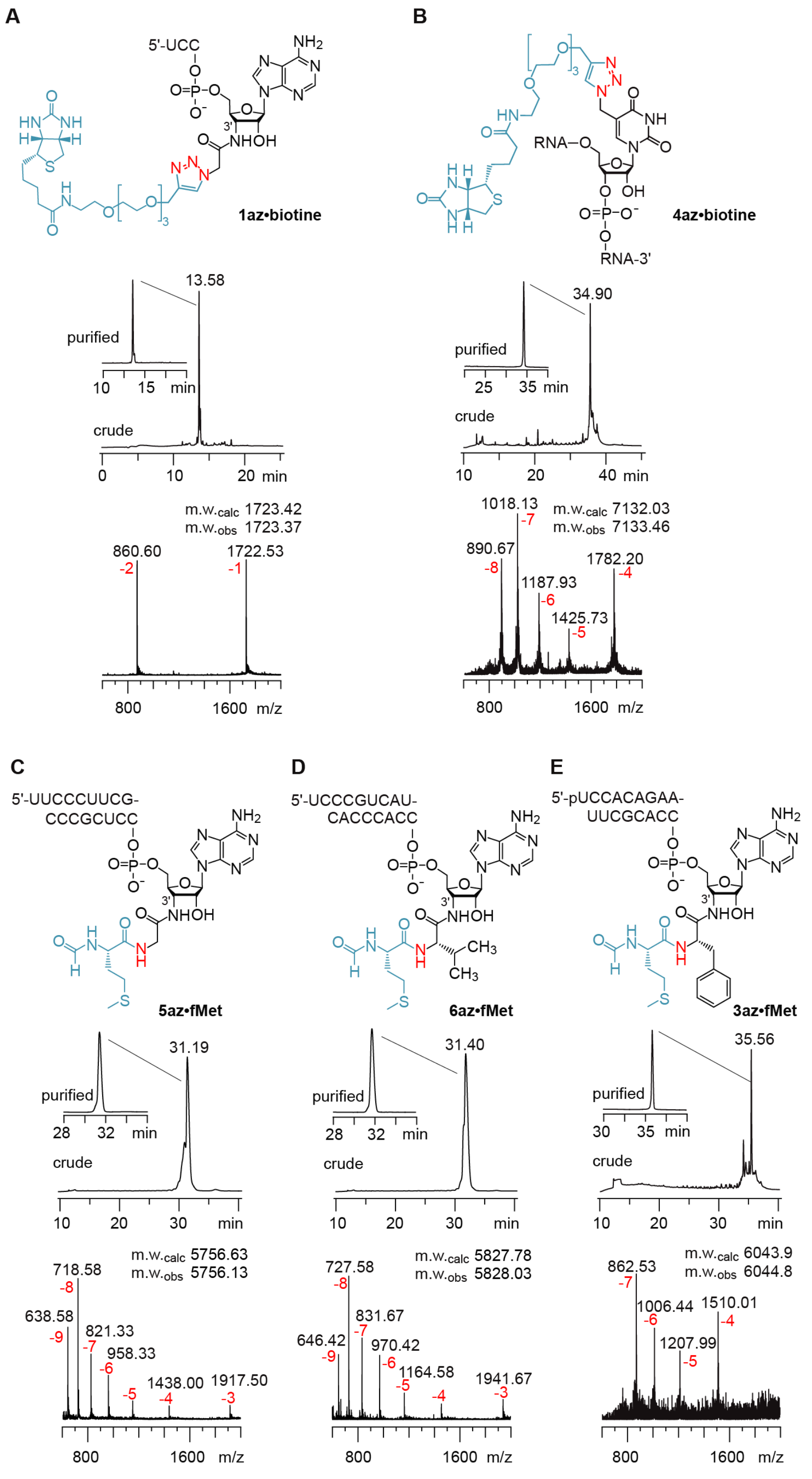

Supporting Figure S4. Applications of azido-RNAs obtained by diazotransfer reaction with FSO_2N_3; anion-exchange HPLC analysis of the reaction mixtures and mass spectrometric characterization of the products of copper(I)-catalyzed alkyne-azide cycloadditions or traceless Staudinger ligations. CuAAC products: (**A**) 4 nt biotinylated glycyl-tRNA mimic, **1az•biotin**; (**B**) 21 nt biotinylated az^{5}U-modified anticodon loop (AL) of *E. coli* tRNA^{Lys}, **4az•biotin**. Traceless Staudinger ligation products: (**C**) 18 nt *N*-formyl-L-methionylglycyl-tRNA mimic **5az•fMet**, (**D**) 18 nt *N*-formyl-L-methionyl-L-valyl-tRNA mimic **6az•fMet**, and (**E**) 18 nt *N*-formyl-L-methionyl-L-phenylalanyl-tRNA mimic **3az•fMet**. For conditions see Methods.

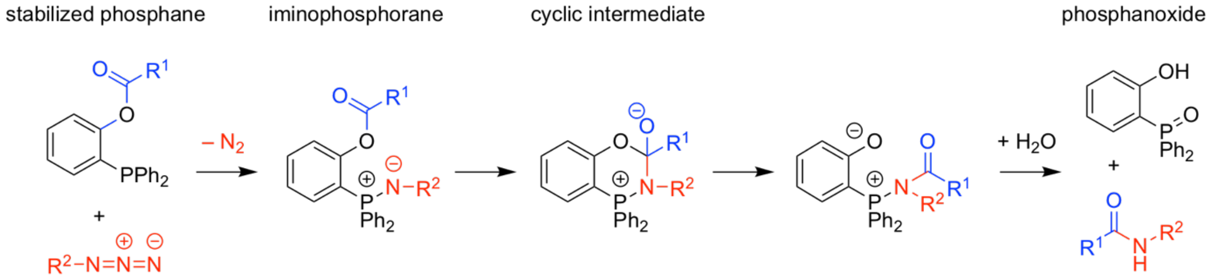

Supporting Figure S5. Proposed mechanism of traceless Staudinger reaction according to reference [5].

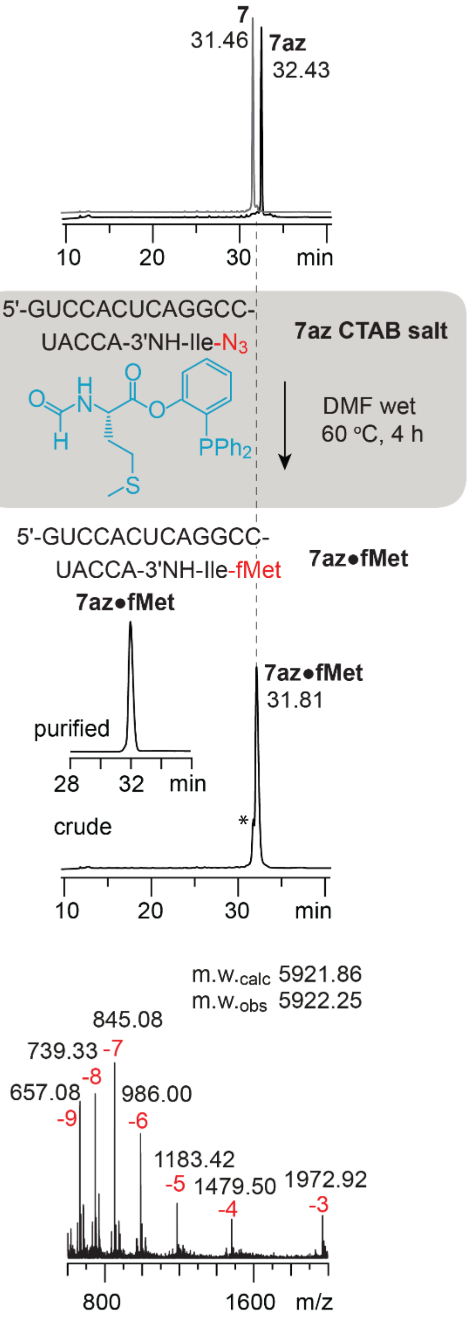

Supporting Figure S6. Extension of Figure 3B. Applications of azido-RNAs obtained by diazotransfer reaction with FSO_2N_3; traceless Staudinger ligation exemplified for a mimic of L-isoleucine charged *E. coli* $tRNA^{Ile}$ (**7az**); analytical anion-exchange (AE) HPLC traces of RNA **7** and **7az** (starting materials), crude and purified conjugates **7az•fMet** (middle), and LC-ESI mass spectrum of purified **7az•fMet** (bottom). The asterisk indicates the minor amounts of a N_3-to-NH_2 reduced byproduct that forms from **7az** under the ligation conditions used. Purification was performed on a semipreparative AE column using gradient elution with a flat slope to separate thee byproduct (for details see Methods above).

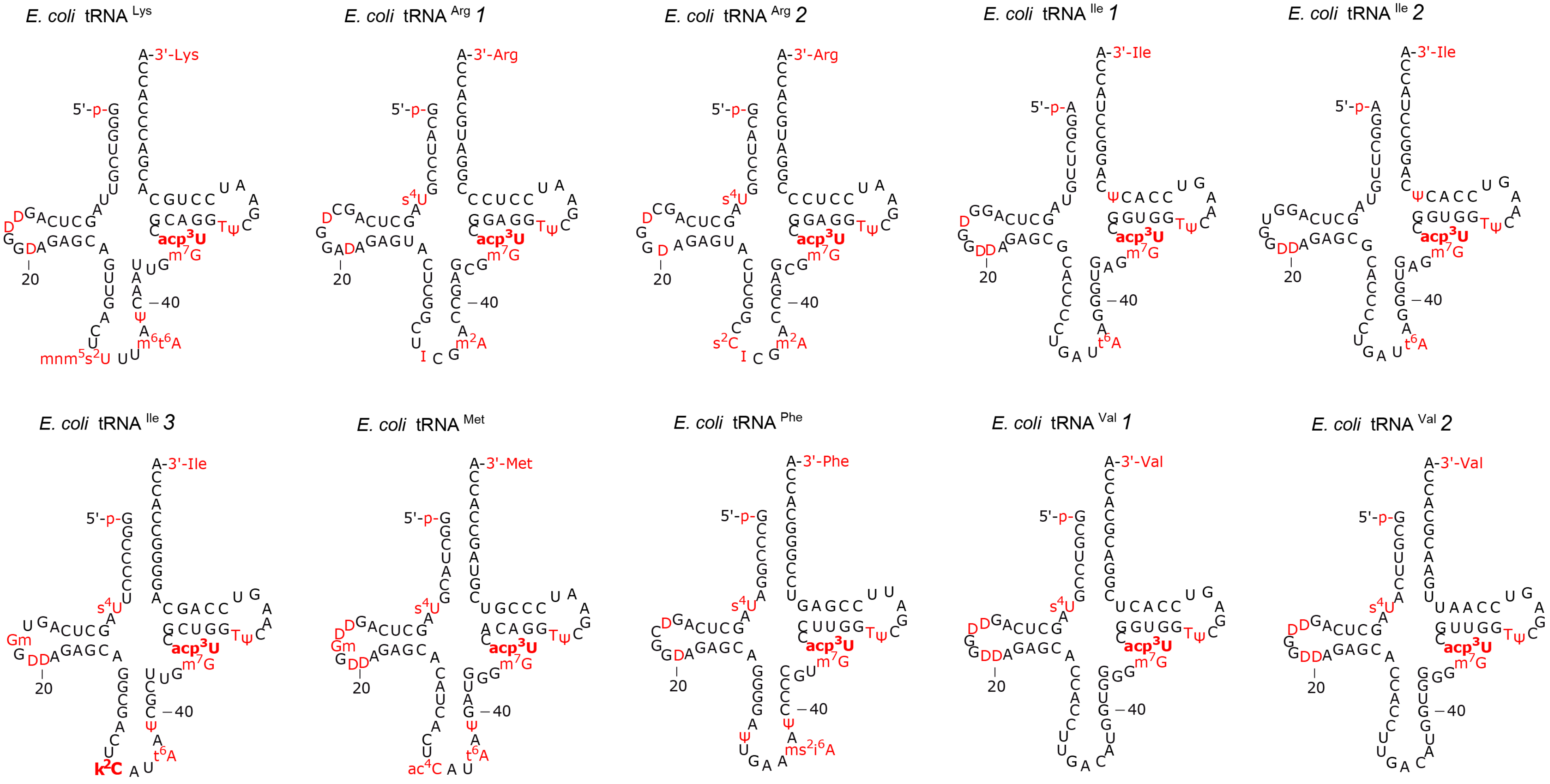

Supporting Figure S7. Clover-leaf structures of *E. coli* tRNAs containing acp^{3}U47 (3-(3-amino-3-carboxypropyl)uridine) and k^{2}C34 (lysidine) [6]. All known genuine tRNA modifications are depicted in red. D – dihydrouridine, mnm^{5}s^{2}U – 5-methylaminomethyl-2-thiouridine, m^{6}t^{6}A – *N*6-methyl-*N*6-threonylcarbamoyladenosine, ms^{2}i^{6}A – 2-methylthio-*N*6-isopentenyladenosine, Ψ – pseudouridine, s^{4}U – 4-thiouridine, I – inosine, m^{2}A – 2-methyladenosine, m^{7}G – 7-methylguanosine, s^{2}C – 2-thiocytidine, t^{6}A – *N*^{6}-threonylcarbamoyladenosine, Gm – 2'-*O*-methylguanosine, ac^{4}C – *N*4-acetylcytidine, p – phosphate.

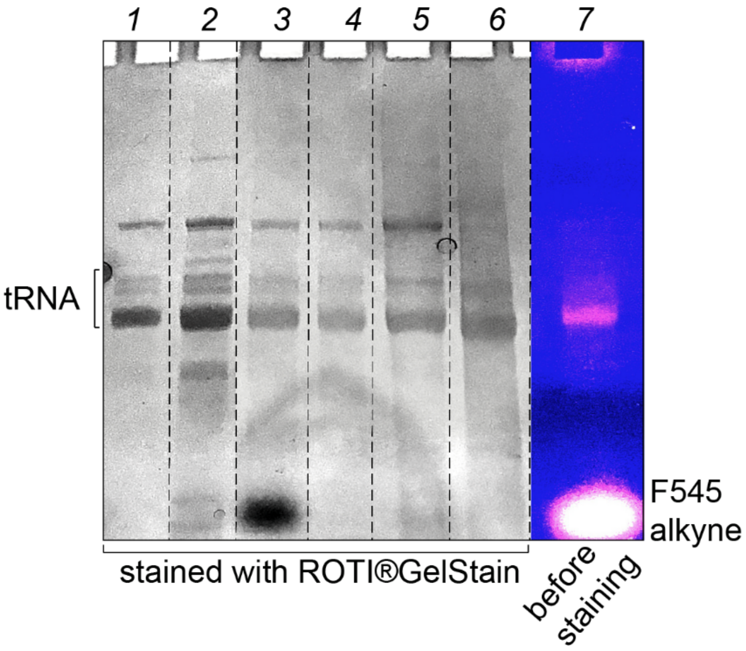

Supporting Figure S8. Analysis of the *E.coli* tRNA mixtures in denaturing 10 % polyacrylamide gel (19:1, 8 M urea). Total tRNA from *E.coli* (lane *1*); *1* after the diazotransfer reaction using FSO_2N_3 (lane *2*); *2* after the CuAAC reaction with F545 alkyne (lane *3*, stained with ROTI®GelStain; and lane *7*, before staining, excited at 254 nm); *2* after the CuAAC reaction with DTB-PEG4-alkyne (lane *4*); a fraction of tRNAs from *4* not bound to the Streptavidin magnetic beads (a mixture of tRNAs without aliphatic amino group, lane *5*); a fraction of tRNAs pulled down (and eluted by excess of biotin) from the Streptavidin magnetic beads (lane *6*).

Supporting Table S1. Overview of amino- and azido-modified oligoribonucleotides and conjugates used in this study.

No.	Oligoribonucleotides[a]	Yields[b] %	RT[c] min	m.w.$_{obs}$[d] amu	m.w.$_{calc}$ amu
**1**	UCCA-3'-NH-**Gly**	46	13.60	1239.67	1241.79
**2**	ACU**nm^{5}U**UUAAU	87	22.90	2790.85	2790.73
**3**	pUCCACAGAAUUCGCACCA-3'-NH-**Phe**	47	34.27	5884.72	5884.67
**4**	CAGUUGACU**nm^{5}U**UUAAUCAAUUG	64	33.42	6649.03	6649.03
**5**	UUCCCUUCGCCCGCUCCA-3'-NH-**Gly**	43	30.46	5596.79	5596.36
**6**	UCCCGUCAUCACCCACCA-3'-NH-**Val**	52	30.65	5669.16	5668.57
**7**	GUCCACUCAGGCCUACCA-3'-NH-**Ile**	72	31.46	5763.10	5762.65
**1az**	UCCA-3'-NH-**Gly**-N_3	98	14.69	1265.67	1265.84
**2az**	ACU**azm^{5}U**UUAAU	98	25.33	2816.88	2816.73
**3az**	pUCCACAGAAUUCGCACCA-3'-NH-**Phe**-N_3	95	35.29	5911.11	5910.70
**4az**	CAGUUGACU**azm^{5}U**UUAAUCAAUUG	96	34.93	6675.40	6675.03
**5az**	UUCCCUUCGCCCGCUCCA-3'-NH-**Gly**-N_3	91	31.61	5622.90	5622.29
**6az**	UCCCGUCAUCACCCACCA-3'-NH-**Val**-N_3	92	31.72	5694.04	5694.57
**7az**	GUCCACUCAGGCCUACCA-3'-NH-**Ile**-N_3	96	32.43	5789.20	5788.65
**1az•biotin**	UCCA-3'-NH-**Gly-biotin**	96	13.58	1723.37	1723.42
**2az•biotin**	ACU**biotin-m^{5}U**UUAAU	98	24.99	3274.40	3274.31
**4az•biotin**	CAGUUGACU**biotin-m^{5}U**UUAAUCAAUUG	78	34.90	7133.46	7132.03
**3az•fMet**	pUCCACAGAAUUCGCACCA-3'NH-**Phe-fMet**	60	35.56	6044.80	6043.90
**5az•fMet**	UUCCCUUCGCCCGCUCCA-3'-NH-**Gly-fMet**	71	31.19	5756.13	5756.63
**6az•fMet**	UCCCGUCAUCACCCACCA-3'-NH-**Val-fMet**	79	31.40	5828.03	5827.78
**7az•fMet**	GUCCACUCAGGCCUACCA-3'-NH-**Ile-fMet**	86	31.81	5922.25	5921.86

[a] Oligonucleotide sequence in 5' to 3' direction and peptide sequence from C to N terminus; [b] determined from areas in HPLC profiles; [c] AE-HPLC retention time (RT); [d] molecular weights m.w. obtained by LC-ESI ion trap mass spectrometry.

Supporting Table S2. Synthetic DNA and 2'-OCH_3 oligonucleotides used as probes for identification of specific *E. coli* tRNAs by Northern blotting.

tRNA (24-46 nt)[a]	Sequence[b]	$m.w._{obs}$[c] amu	$m.w._{calc}$ amu
**tRNA^{Lys}**	5'-d(CGACCAATTGATTAAAAGTCAACTGCTCT)	8844.73	8844.85
**tRNA^{Arg} 1** and **tRNA^{Arg} 2**	5'-d(TCGCTCGGTTCGTAGCCGAGTAC)	7032.11	7031.61
**tRNA^{Ile} 1** and **tRNA^{Ile} 2**	5'-d(TCTCACCCTTATCAGGGGTGCGC)	6991.55	6991.59
**tRNA^{Ile} 3**	5'-m(CCAAGCGAUUAUGAGUCGCCUGCUC)dT	8588.52	8588.70
**tRNA^{Met}**	5'-d(TCCCATCATTATGAGTGATGTGC)	7029.85	7029.64
**tRNA^{Phe}**	5'-m(CACGGGGAUUUCAAUCCCCUGCUC)dT	8525.68	8525.60
**tRNA^{Val} 1** and **tRNA^{Val} 2**	5'-d(TCCCCACCATGTCAAGGTGGTGC)	7000.73	7000.60
Reference **tRNA^{Gly}**	5'-d(TCCCGACCTTGGCAAGGTCGTGC)	7016.78	7016.60

[a] See **Supporting Figure 7** for sequences and modifications of *E. coli* tRNAs. [b] dN – deoxyribonucleotide, mN - 2'-(*O*-methyl)ribonucleotide. [c] Reversed-phase LC-ESI ion trap mass spectrometry (see Methods).

References

1. Steger J., Micura R. Functionalized polystyrene supports for solid-phase synthesis of glycyl-, alanyl-, and isoleucyl-RNA conjugates as hydrolysis-resistant mimics of peptidyl-tRNAs. *Bioorg. Med. Chem.* **19**, 5167-5174 (2011).
2. Meng G., Guo T., Ma T., Zhang J., Shen Y., Sharpless K.B., Dong J. Modular click chemistry libraries for functional screens using a diazotizing reagent. *Nature* **574**, 86–89 (2019).
3. a. El-Sagheer A. H., Brown, T. New strategy for the synthesis of chemically modified RNA constructs exemplified by hairpin and hammerhead ribozymes. *Proc. Natl. Acad. Sci. USA* **107**, 15329-15334 (2010); b. Chan T.R., Hilgraf R., Sharpless K.B., Fokin V.V. Polytriazoles as copper(I)-stabilizing ligands in catalysis. *Org. Lett.* **6**, 2853–2855 (2004).
4. McCown P.J., Ruszkowska A., Kunkler C.N., Breger K., Hulewicz J.P., Wang M.C., Springer N.A., Brown J.A. Naturally occurring modified ribonucleosides. *Wiley Interdiscip. Rev. RNA* **11**, e1595 (2020).
5. a. Bednarek C., Wehl I., Jung N., Schepers U., Bräse S. The Staudinger Ligation. *Chem. Rev.* **120**, 4301–4354 (2020); b. Soellner M.B., Nilsson B.L., Raines, R.T., 2006. Reaction mechanism and kinetics of the traceless Staudinger ligation. *J. Am. Chem. Soc.* **128**, .8820–8828 (2006).
6. Jühling F., Mörl M., Hartmann R.K., Sprinzl M., Stadler P.F., Pütz J. tRNAdb 2009: compilation of tRNA sequences and tRNA genes, *Nucleic Acids Res*. **37**, D159-D162 (2009).
